# Role of Program Curriculum in Building Social Skills and Sports Coaching in Academic and Career Development Under Sports Humanities and Sociology

**DOI:** 10.3389/fpsyg.2022.852331

**Published:** 2022-03-10

**Authors:** Zhenglu Jiang, Jiesen Yin

**Affiliations:** ^1^Physical Education Department, Jiangnan University, Wuxi, China; ^2^Department of Physical Education, Wuxi Institute Technology, Wuxi, China; ^3^Graduate School Division José Rizal University, Mandaluyong, Philippines

**Keywords:** social skills coaching, sports coaching, academic development, career development, organizational climate

## Abstract

This study focused on the role of program curriculum in building social skills and sports coaching in academic and career development in terms of sports humanities and sociology. Social skills coaching and sports coaching for the students are two significant factors that need to be considered by the universities around the globe to improve the organizational climate, which ultimately lead to better student’s career and academic development. This study utilized data from 308 members of the sports federation enrolled in different programs in the universities in China through a questionnaire for investigating such influencing factors. The data were analyzed using the structural equation modeling (SEM) technique using SmartPLS. This study found a substantial relationship of social skills with academic development and organizational climate. The relationship of sports coaching with organizational climate and career development is also discussed in this study. Furthermore, the organizational climate acts as a partial mediator between sports coaching and career development. However, this study revealed that organizational climate had an insignificant relationship with academic growth and organizational climate did not mediate between social skills coaching and educational development among students. While hiring the sports personnel, universities must consider the coach’s competency using effective methods such as physical tests, preassessments, and psychological tests. Second, the universities in China have to develop a culture of social skills and sports to improve academic and career development.

## Introduction

Social skills coaching and sports coaching are the constituents of organizational processes, leading to achievement in academic development and career development. The organizational climate is also an essential part of the organizational processes. It could mediate the relationships between coaching social skills, sports skills, and academic and career development. These relationships were studied before in different perspectives on determining the youth outcomes in sports organizations (Lower-Hoppe et al., 2020). Sport is an effective tool, but it must be purposefully structured and enabled to achieve kids’ developmental outcomes. The role of the youth sports coach is critical to the effectiveness of using sport as a social intervention to achieve youth developmental milestones. The role of a sports coach extends beyond that of a sport-skills instructor (Newman et al., 2016). Mentors, instructors, father figures, activists, relative’s dalliances, and community activists are all common examples of prominent figures. Furthermore, sports coaches are primary workers and influencers, responsible for creating an emotionally and physically safe environment, giving chances for developing skills and purposefully directing sports interactions toward developmental objectives (Gould et al., 2012; Camiré et al., 2013).

Many formerly unconnected fields, such as social work, psychology, sport management, and physical education, are becoming conferred collaborators in examining and analyzing the influencing function of sports coaches due to their crucial role within youth sports culture. Although cross-disciplinary initiatives, most coaches lack professional coaching training or education, particularly when it comes to young development methods (Gould and Carson, 2008; Camiré et al., 2011). Consequently, sports coaches are frequently allowed to establish their coaching ideas and techniques (Camiré et al., 2014). The coaching sports skills and social skills are mainly based on positive youth development models, which is the model of coaching life skills and the major contributor is the sport (Gould and Carson, 2008).

According to the social cognitive theory, student’s academic accomplishment abilities are the consequence of constant, reciprocal connections among behavior, such as social skills, the surrounding world, cognition, and other internal factors that might alter perceptions and behaviors. Student’s socialization appears to either help or impede their understanding. Their academic accomplishment can impact their knowledge and influence their behavior and provide an opportunity to build social skills and connections (Miles and Stipek, 2006). Scientists usually utilize grades to assess a student’s academic progress. Furthermore, studies have shown that scores are impacted by various elements, notably social skills, contributing to academic subject knowledge (Lekholm and Cliffordson, 2008). However, after adjusting for high IQ scores, culture, academic behavior, and even coach preferences, prosocial behavior predicts student’s results. The extent to which students act in class appears to have an indirect impact as how well they learn and perform. When it comes to assigning grades to children, social skills are crucial. The results of object tests have a weaker relationship with student’s social abilities than subjective instructor judgments. Subjective teacher evaluations show higher gender inequalities than objective examination scores (Lekholm and Cliffordson, 2008; DiPrete and Jennings, 2012; Cornwell et al., 2013).

Teachers also require students to have specific skills to do schoolwork, such as self-control and teamwork, throughout kindergarten to high school. The capacity of students to achieve such standards affects their academic or social school experiences. Learners who acquired good social skills in preschool were much more effective in their duties as learners and in learning specific social entrance tasks informal education, such as attending, following instructions and listening to activities (Lane et al., 2006; Lynne Lane et al., 2007). Furthermore, these children were better able to participate in class, have more favorable views regarding education, and did better overall (Ladd et al., 2006; Konold et al., 2010). In organizational behavior theory, organizational climate and process are essential antecedents of organizational performance. Organizational climate, in general, indicates how people of an organization view the organization’s social environment. Furthermore, it demonstrates the ordinary meaning that organizational members attach to the events, rules, practices, and procedures they encounter as well as the behaviors they perceive rewarded, encouraged, and anticipated (Jordan et al., 2009; Ehrhart and Raver, 2014).

Regardless of the organization, workers spend a significant amount of time at work, committing personal and group effort to fulfill the goals set out by the entity’s direction and management; hence, the work environment is critical to a person’s well-being (Jordan et al., 2009; Ehrhart and Raver, 2014). The difficulty in distinguishing between the organizational environment created by employees and the organization’s employee welfare necessitates focusing attention and prioritizing an organizational study of sports clubs. However, knowledge and research have become a vital aspect of the organizational phenomena due to the organization’s employees significant effect on the entity’s overall behavior. On the other hand, the organizational climate is one of the most significant aspects to consider, since it has a direct positive association with the worker’s ultimate performance and, as a result, the organization’s overall success (Ahmad et al., 2018; Berberoglu, 2018). Amidst the cultural, financial, and sports activities significance of sports clubs in society, few studies have analyzed the organizational climate in the associative sports field and sometimes even very few pragmatic applications or management implications have indeed been supplied from research and academic fields (Kojour et al., 2017; Escamilla-Fajardo et al., 2020).

Despite the fact that many longitudinal studies have looked at the effects of student’s social skills on both the current and future academic success, there are still major gaps in the literature. To begin with, most researchers have explained academic accomplishment in terms of reading and mathematical ability, with minimal study on the relationship between social skills and academic progress. Second, just a few studies have looked at students from various grades. Rather, only kids from one or two grades were assessed at various intervals in time. Third, the link between social skills and academic development has received little consideration (Gustavsen, 2017). Moreover, the connection between coaching social skills to students also received little attention. Some researchers found social skill coaching and sport coaching as organizational processes and found the impact of organizational climate on youth outcomes (Lower-Hoppe et al., 2020). While outcomes were more generalized than specific, a gap arose in defining the outcomes. According to organizational behavior theory, an organization’s procedures indirectly influence performance through organizational climate. Still, there is no direct effect on performance due to the indirect effect. Moreover, organizational climate was assessed for its direct effects on outcomes, while it could be analyzed in indirect way mediating the relationship of coaching social skills and sports skills with outcomes such as academic and career development of students. Based upon these gaps and significance of the factors contributing program curriculum, this research was designed addressing the research questions. This study revolved around following objectives as:

1. *To assess the role of coaching social and sports skills on career development.*

2. *To evaluate the role of sports coaching and social skills coaching on academic development.*

3. *To explore organizational climate as a mediator between skill coaching and socioacademic development.*

## Review of Literature and Hypothesis Development

This framework is based on three important theories that analyze the relationships between coaching social skills, sports coaching, organizational climate, and academic and career development.

### Experiential Learning Theory

Teaching or coaching of social skills is a part of organizational processes based on experiential learning theory suggested by Jones et al. (2017). Experiential learning theory was given by Kolb (1984) providing experience obtained through coaching that leads to learning and the development. Experiential learning, as the title indicates, is based on personal experience. David Kolb, a psychotherapist, created the idea after being inspired by the research of many other theorists such as Dewey, Lewin, and Piaget. Another sort of learning, according to Kolb, is “the process by which knowledge is formed via the transformative learning.” The process of comprehending and changing an experience yields knowledge. According to Kolb, a variety of circumstances can impact preferred learning methods. Adaptive abilities, career choice, present job function, educational specialty, and personality type are some of the elements he has discovered (Kolb, 1984). Some of the researchers suggested the theory as basis for devising program curriculum of social skill learning in the context of youth development programs (YDPs) through sports (Jones et al., 2017). School developed a three-phase procedure to assist experiential learning: briefing to define the experience, action to enable the expertise, and reflection to learning from past on the expertise (Lower-Hoppe et al., 2020). This theory allowed us to utilize coaching social skill in career and academic development by mediating organizational climate.

### Social Cognitive Theory

The social cognitive theory outlines the method of learning through observation and imitation. The theory refers to a psychological model of activity based on Bandura’s research. It must have been created with such an emphasis on the acquisition of new behaviors in mind and it continues to emphasize that learning takes place in a social setting, with most of what is acquired coming through observation. Because it focuses on how mental cognitive aspects are engaged in learning, social cognitive theory is sometimes referred to as a bridge between classical learning theory, such as behaviorism and the mental process (Bandura, 1977). As per Bandura, most social learning is based on imitation, a process known as observational learning and the best approach to understanding how learning happens is to study behavior modification.

Because learners must pay attention, develop and store mental representations of what they see, recover these representations from memory later, and use them to guide action, observational learning is considered a cognitive kind of learning (Sigelman and Rider, 2009). According to Bandura, observational learning may be summed up in two statements: most of the human learning is based on seeing and copying the actions of others or symbolic models such as fictitious characters in television shows and literature. Imitative behavior can be regarded an operant from a Skinnerian perspective, which is a response that is not generated by any known or evident stimuli. In basic words, the person choose whether or not to respond. Imitative activities become more likely when they result in positive consequences or eliminate or prevent negative contingencies. In operant conditioning terms, a reinforcer enhances the likelihood that a response (i.e., behavior) will occur (Sigelman and Rider, 2009). The application of social cognitive theory as basis of sports coaching has been practiced in past (Jones et al., 2017).

### Organizational Behavior Theory

According to organizational behavior theory, an organization’s procedures have an indirect influence on performance through organizational climate, but there is no direct effect on performance as a result of the indirect effect (Lower-Hoppe et al., 2020). Organizational behavior theory is the basis for studying situations in youth sport programs that may have an impact on young people’s development. Youth accomplishments are seen as a key indication of organizational effectiveness among the community youth sports groups (Kaplan, 2001; Anderson-Butcher et al., 2014). This objective is comparable to how most non-profit sports organizations evaluate their effectiveness by looking at their members. More specifically, when members of an organization engage in targeted behaviors, such as organizational citizenship or demonstrate achievement of intended outcomes, such as learning and development that are aligned with the organization’s mission and goals, the organization has evidence of its performance (Ehrhart and Raver, 2014).

Organizational performance has been postulated due to organizational environment and process in organizational behavior theory. Youth outcomes, such as organizational performance, are impacted by youth participants’ impressions of the program environment, such as organizational climate, in the setting of a youth athletic program. The project’s policies and procedures or organizational process shapes the project’s climate. As a result, a community youth sports organization’s climate, policies, and practices should potentially impact juvenile outcomes (Ehrhart and Raver, 2014). A few researchers assessed the organizational climate under the lenses of organizational behavior theory (Wheeler, 2000; Daily et al., 2013; Berberoglu, 2018). So, based on these situations of organizational behavior in managing the organizational climate for academic and career development, this research was formulated.

### Association of Coaching Social Skills With Organizational Climate and Academic Development

There are several reasons to believe that sports and physical activities provide a good environment in which to develop these abilities. Numerous youngsters already engage in sports activities, whether individually or in groups and structured or unstructured, but these settings provide numerous chances to grow essential skills such as teamwork as well as collaboration, compassion and prosocial behavior, making plans, and solving problems. According to the Sports and Fitness Industry Association, which counts participation in 120 sports, recreation, and fitness activities in the United States, more than 70% of children aged 6–12 years participate in particular team sports at least 1 day each year (Hurd and Deutsch, 2017). Academic development in school necessitates mastery of certain abilities such as social and academic work.

A contrast is drawn in behavior between the ideas of social skills and social competency, which are defined differently in the literature. Social awareness involves the cognitive knowledge of social skills and how to apply them in interactions with other students, whereas social skills are the actual behaviors that students accomplish in specific social contexts (Gresham et al., 2010; Wentzel, 2015). Student’s social skills were defined in this study as a significant collection of acquired behaviors that facilitate pleasant interactions with people in their surroundings. Social skills are demonstrated by behavior and it is typical to categorize teacher-rated social skills into three categories: collaboration, self-control, and assertiveness (Gustavsen, 2017). There are several forms of social skills deficiencies, which are defined as difficulties in learning or performing social behaviors. When acquiring deficits are caused by a student’s lack of specific social skills, it means that the student does not know how to perform the targeted social skill and, thus, cannot solve problems. When performance deficits are caused by a student knowing how to perform, but not displaying it appropriately, the student cannot solve problems (Gresham et al., 2010).

Student’s behavior or interactions with classmates and teachers are the sole ways for researchers to investigate social skills in schools. Student’s social skills may be influenced by their educational environment, but they may also choose whether to use their social skills positively or negatively. Individual and interpersonal results are a product of student’s personal efforts as well as interactions with classmates and teachers (Wentzel, 2015). Longitudinal studies of the influence of social skills on academic success have yielded varied results. Some studies have identified a substantial link between social skills and subsequent academic success (Caprara et al., 2000), whereas others have indicated that social skills are not a major predictor of later academic achievement (Claessens et al., 2009). A meta-analysis of a broad sample of kids from kindergarten through high school found that universal socioemotional learning programs in schools resulted in an increased prosocial behavior, social skills, and success (Claessens et al., 2009). This could lead to a linear relationship between teaching social skills in sports context with academic development and the organizational climate being a mediator between the both could be developed (Lower-Hoppe et al., 2020). So, the following hypothesis were developed.

***H_1_***: *Social skills coaching has an impact on academic development.*

***H*_2_**: *Social skills coaching has an impact on organizational climate.*

### Association of Sports Coaching With Organizational Climate and Career Development

Sport coaches are trusted by parents, athletes, and athletic organizations to help athletes improve both on and off the field. Coaches are critical in supporting good youth development via sport, since they impact the sports atmosphere. Positive youth development is more likely when sport coaches intentionally establish mastery-oriented environments that strive to develop players beyond athletic skills and strategies (Feltz et al., 1999; Cope et al., 2013; Camiré, 2015; Strachan et al., 2016; Holt et al., 2017). Coaches must stress the purpose of sport as learning from mistakes while appreciating working hard for personal growth in order to create a mastery-oriented atmosphere favorable to good young development.

Coaches should concentrate on their athletes growth rather than their achievement and players should be judged against self rather than others. Coaches must ensure that all the athletes are valued and appreciated. Coaches may help athletes grow even more by introducing intentional lessons into their programs that teach life skills such as emotional control, cultural competency, personal responsibility, and interpersonal skills. Finally, coaching techniques such as role modeling, creating solid connections, employing compassionate conversation, and positive thinking can help child athletes develop positively (Gould and Carson, 2008; Light, 2010; Camiré et al., 2012; Falcão et al., 2012). Unfortunately, athlete growth via athletics is not always favorable. Coaches impact performance-oriented athletes in the same way as they influence mastery-oriented surroundings, presumably owing to societal standards of winning at all the costs (Shields and Bredemeier, 2010; Neely and Holt, 2011). Impact of sports coaching could be further analyzed for organizational climate as suggested by Lower-Hoppe et al. (2020) and career development of students after completing the education so, we devised the following hypothesis.

***H*_3_**: *Sports coaching has an impact on organizational climate.*

***H*_4_**: *Sports coaching has an impact on career development.*

### Impact of Organizational Climate on Organizational Processes and Its Mediating Role

A display of the culture, a collection of attitudes, sentiments, and actions that characterize corporate life and an organizational reality with an objective idea are all examples of organizational climate. The organizational climate refers to the core organizational components such as attitudes and understandings among the organization’s members. Because the climate is dependent on individual viewpoints, it varies rapidly and influences the behavior of the organization’s members. The environment in which individuals work may impact their ability to be creative and innovative. Having a creative climate that is in sync with the organization’s culture creates an atmosphere that boosts organizational power (West and Farr, 1989; Ekvall, 1996; Nasurdin et al., 2006). Because they come from separate theoretical traditions, the phrases “organizational climate” and “organizational culture” are frequently viewed as independent ideas. However, they contain many overlapping components. Climate, according to Stringer, is a subset of the corporate culture. Culture refers to all the businesses deeply ingrained value systems, which are difficult to modify. The context and milieu in which a work group performs are influenced by organizational strategy, the macroenvironment, organizational configurations, and historical influences. Such “cultural” impacts emerge from outside the work group and outside the manager’s direct control (Denison, 1996). The environment of a work group may be comparable to or distinct from that of the organization as a whole. High-performing work groups can occasionally be found in companies that are suffering from dwindling budgets or a lack of senior leadership. Even if an organization’s atmosphere is less than ideal, a manager’s leadership and management skills may produce a favorable work climate and great results inside a work group. Regardless of a manager’s level, efforts to improve the workgroup’s atmosphere can lead to great employee performance and outcomes (Perry et al., 2005).

Along with these contributions of organizational climate in different organizational processes, the organizational climate had been studied in mediating and moderating the relationships of different organizational processes (Imran and Anis-ul-Haque, 2011; Al Ghazo et al., 2019; Mehralian et al., 2020). Moreover, direct impacts were studied by Ehrhart and Raver (2014) and Lower-Hoppe et al. (2020) suggested indirect role of organizational climate for future studies. Hence, we developed the following hypothesis for testing.

***H*_5_**: *Organizational climate has an impact on academic development.*

***H*_6_**: *Organizational climate has an impact on career development.*

***H*_7_**: *Organizational climate mediates the relationship between social skill coaching and academic development.*

***H*_8_**: *Organizational climate mediates the relationship of sports coaching and career development.*

**FIGURE 1:**
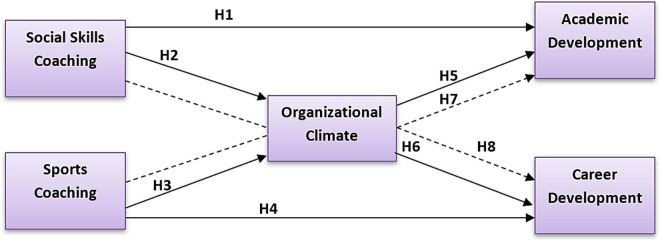
Conceptual framework.

A following conceptual model (Figure 1) has been formed based on the above literature and hypothesis.

## Methodology

This study followed the deductive approach with a quantitative research design to test the hypothesis developed to examine the impact of predicting variables on outcome variables. This approach has been used to reduce biases. A self-administered survey was used to collect the data for this quantitative study. This study population included members of the sports federation enrolled in different programs in the Chinese universities. Purposive sampling was used to select the sample for the survey. This sampling technique helped the research take data from only those students involved in any sports. The survey questionnaires were distributed and collected after a week from the study participants. A total of 308 usable questionnaires were received, which were then analyzed. The unit of analysis for this study was the sports federation members enrolled in different programs in the universities in China.

### Instrument Development

The questionnaire was used as the survey instrument for this study. The questionnaire included items for each variable. This study consisted of five variables, social skill coaching and sports coaching; two dependent variables, namely, academic development and career development; and one mediating variable, namely, organizational climate. This study adopted a scale from the past studies conducted in a similar context. A six-item scale for social skill coaching and a five-item scale for sports coaching were also adopted from a study by Lower-Hoppe et al. (2020). A four-item scale for academic development and a three-item scale for career development were adopted from a study by Lee (2020). A 14-item scale for the organizational climate was adopted from a study by Perry et al. (2005). A five-point Likert scale (from 1 being strongly disagreed to 5 being strongly agreed) was used to collect the responses from the study participants.

### Demographics Details

Out of the sports federation members (overall 308), there were 59.09% males and 40.09% females. The participants below 20 years were 36.36, 31.49% were in the age bracket of 21–25 years, 23.38% were between the age of 23.38%, and the participants who were 31 years were above 8.77%. The participants with Bachelor’s degrees were 45.80%, Master’s degree holders were 37.09%, and the respondents with Ph.D. or other degrees were 17.09%. Table 1 below shows the demographics details.

**TABLE 1:** Demographics analysis.

Demographics	Frequency	Percentage
**Gender**		
Male	182	59.09%
Female	126	40.09%
**Age (years)**		
Below 20	112	36.36%
21 to 25	97	31.49%
26 to 30	72	23.38%
31 and above	27	8.77%
**Education**		
Bachelors	142	46.10%
Masters	115	37.33%
Ph.D. or others	51	16.55%

*N = 308.*

## Data Analysis and Results

Data analysis was carried out using SmartPLS version 3.3.3 software. This software is widely used for structural equation modeling (SEM). SmartPLS helps to analyze the data in two stages. The first stage includes a measurement model investigating the data validity and reliability. Factor loadings for each item, average variance extracted (AVE), the heterotrait-monotrait (HTMT) ratio, and Fornell and Larcker tests are performed to examine the validity of the data. In contrast, data reliability is determined using the Cronbach’s alpha and composite reliabilities. The second stage involves a structural model in which this study is tested based on the hypothesis that depends on the values of *t*-statistics, sig-values, the sample mean, and SD.

### Measurement Model

**FIGURE 2:**
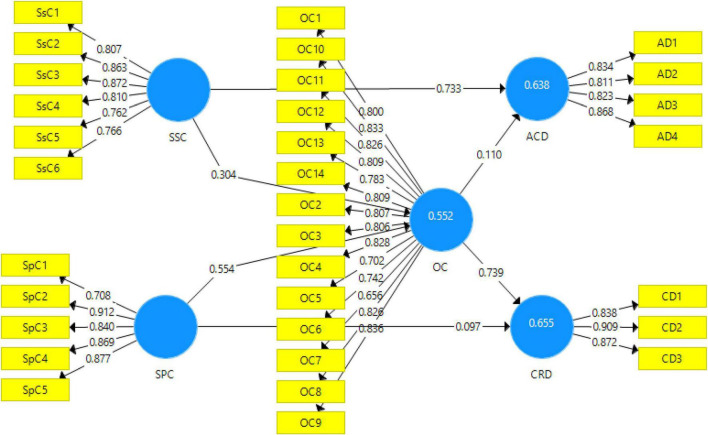
Output of measurement model algorithm. SSC, social skills coaching; SPC, sports coaching; ACD, academic development; CRD, career development; OC, organizational climate.

The measurement model algorithm of this study is given in Figure 2, explaining how much the study’s independent variables contributed toward dependent variables.

Table 2 shows each item’s factor loadings and variance inflation facto (VIF) values. Factor loadings are used to explain the contribution made by each item of the variable and use multiple items to measure a required and desirable variable (Lambert et al., 1991). According to Lambert et al. (1991), factor loading values have been categorized into three different classes, i.e., undesirable (value < 0.3), fair (value > 0.5), and highly desirable (value > 0.7). The benchmark values for factor loadings were 0.5 and above for this study. For this study, all the factor loading values are above the set benchmark of 0.5. Hence, a significant contribution was made by each item toward this study.

**TABLE 2:** Factor loadings, reliabilities, and average variance extracted (AVE).

Variables	Factor loadings		Cronbach alpha	Composite reliability	AVE
Social skill coaching	SSC1	0.807	0.879	0.909	0.625
	SSC2	0.863			
	SSC3	0.872			
	SSC4	0.810			
	SSC5	0.762			
	SSC6	0.766			
Sports coaching	SPC1	0.708	0.898	0.925	0.712
	SPC2	0.912			
	SPC3	0.839			
	SPC4	0.869			
	SPC5	0.877			
Academic development	ACD1	0.834	0.839	0.892	0.674
	ACD2	0.811			
	ACD3	0.823			
	ACD4	0.868			
Career development	CRD1	0.838	0.823	0.895	0.739
	CRD2	0.909			
	CRD3	0.872			
Organizational climate	OC1	0.801	0.954	0.959	0.627
	OC2	0.834			
	OC3	0.826			
	OC4	0.808			
	OC5	0.782			
	OC6	0.809			
	OC7	0.808			
	OC8	0.807			
	OC9	0.829			
	OC10	0.702			
	OC11	0.742			
	OC12	0.656			
	OC13	0.826			
	OC14	0.837			

Table 2 shows the alpha reliabilities and AVE values. Alpha reliabilities include the Cronbach’s alpha, which should be higher than 0.7 (Dar et al., 2022). The Cronbach’s alpha values for each variable are above the threshold (ranging from 0.823 to 0.954), indicating that the variables were highly reliable. Moreover, unlike the Cronbach’s alpha, equal loading for a construct is not included in composite reliability. The value of composite reliability ranges from 0 to 1 and it is also divided into three categories: fair reliability (value of 0.6), satisfactory reliability (value from 0.6 to 0.7), and highly satisfactory reliability (value from 0.7 to 0.9). Table 2 depicts that the value of composite reliability for every construct is more than 0.6; therefore, the composite reliability is considered to be satisfactory. Furthermore, AVE examines the existence of convergent validity and its value must be greater than 0.5 (Avotra et al., 2021). Table 2 below shows that the value of AVE for every construct is more than 0.5, indicating the presence of convergent validity.

The HTMT ratio and Fornell and Larcker criterion are two tests that are commonly used to examine the discriminant validity of the variables under study. Discriminant validity explains whether one construct is different from the other construct or not. Considering the HTMT ratio for determining the discriminant validity between variables, the value of the HTMT ratio must be below 0.9 (Franke and Sarstedt, 2019). Table 3 shows that the value of the HTMT ratio of every construct is below 0.90; thus, discriminant validity exists between the variables. Similarly, to determine the discriminant validity of the construct using Fornell and Larcker criterion, the square root of every variable’s AVE must be more than the highest correlation of the construct with the other latent variable. The result of Fornell and Larcker criterion has been shown in Table 4. Table 4 shows that the value on top of each column is higher than the following values indicating that discriminant validity exists between the variables.

**TABLE 3:** The heterotrait-monotrait (HTMT) ratio.

	ACD	CRD	OC	SPC	SSC
**ACD**					
CRD	0.532				
OC	0.571	0.898			
SPC	0.382	0.665	0.724		
SSC	0.892	0.599	0.595	0.488	

*SSC, social skills coaching; SPC, sports coaching; ACD, academic development; CRD, career development; OC, organizational climate.*

**TABLE 4:** Fornell and Larcker criteria.

	ACD	CRD	OC	SPC	SSC
ACD	0.834				
CRD	0.457	0.874			
OC	0.517	0.807	0.792		
SPC	0.350	0.609	0.692	0.844	
SSC	0.794	0.527	0.555	0.454	0.814

*SSC, social skills coaching; SPC, sports coaching; ACD, academic development; CRD, career development; OC, organizational climate.*

The *R*^2^ values for the dependent variables, i.e., academic development and career development, are 63.8 and 65.5%, respectively, and the *R*^2^ value for the mediating variable, i.e., organizational climate, came out to be 55.2%.

### Structural Model

**FIGURE 3:**
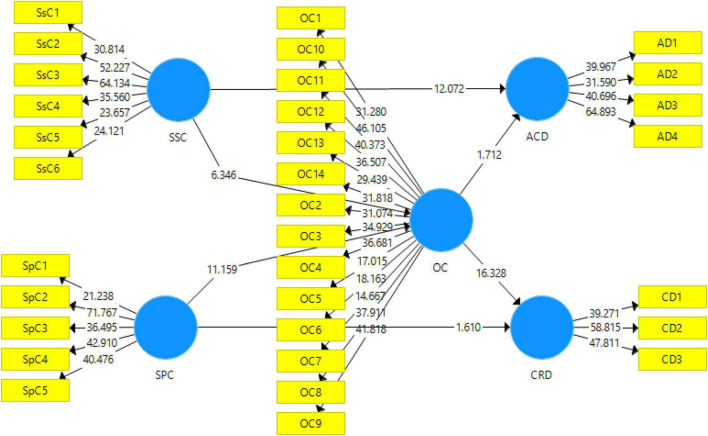
Output of structural model bootstrapping.SSC, social skills coaching; SPC, sports coaching; ACD, academic development; CRD, career development; OC, organizational climate.

As explained above, once the measurement model’s criteria are completed, the structural model is examined. The data analysis of this study requires a thorough examination of the structural model. This involves analyzing the direct relationship between the study variables through partial least squares (PLS)-SEM bootstrapping (see Figure 3). The model includes values of coefficients and *p*-values. A 95% bias-corrected bootstrap was adopted for this study to analyze the direct relationship between the variables (Yingfei et al., 2021). Additionally, this section also includes *t*-statistics, SE, and path coefficients to examine the study’s hypothesis (see Table 5).

**TABLE 5:** The direct effects of the variables.

	Hypothesis	Original sample (O)	Sample mean (M)	Standard deviation	*T* Statistics	*P* Values	Result
SSC -> ACD	H_1_	0.733	0.737	0.061	12.072	0.000***	Accepted
SSC -> OC	H_2_	0.304	0.301	0.048	6.346	0.000***	Accepted
SPC -> OC	H_3_	0.554	0.555	0.050	11.159	0.000***	Accepted
SPC -> CRD	H_4_	0.097	0.099	0.060	1.610	0.108	Rejected
OC -> ACD	H_5_	0.110	0.109	0.064	1.712	0.087	Rejected
OC -> CRD	H_6_	0.739	0.736	0.045	16.328	0.000***	Accepted

***p < 0.001. SSC, social skills coaching; SPC, sports coaching; ACD, academic development; CRD, career development; OC, organizational climate.

Tables 5, 6 show the results of each hypothesis using PLS-SEM algorithms and PLS-SEM bootstrapping. The inner model has been analyzed with the help of indicators such as *p*-value, *t*-statistics, the original sample mean, and SD. Table 5 shows that all the outer loadings, except one, are significant, as the value of *t*-statistics came out to be more than 1.96.

**TABLE 6:** The indirect effects of the variables.

	H	Original sample (O)	Sample mean (M)	Standard deviation (STDEV)	*T* Statistics (|O/STDEV|)	*P* Values	Result
SSC -> OC -> ACD	H_7_	0.033	0.034	0.021	1.574	0.116	Rejected
SPC -> OC -> CRD	H_8_	0.409	0.408	0.041	10.050	0.000***	Accepted

***p < 0.001. SSC, social skills coaching; SPC, sports coaching; ACD, academic development; CRD, career development; OC, organizational climate.

The study’s first hypothesis (H1) states that social skill coaching impacts academic development. The result of this hypothesis shows that *t*-statistics = 12.07 with *p*-value = 0.000 (*p* < 0.05), thus this hypothesis has been accepted. The study’s second hypothesis (H2) states that social skill coaching impacts organizational climate. The result of this hypothesis shows that *t*-statistics = 6.34 with *p*-value = 0.000 (*p* < 0.05), thus this hypothesis has also been accepted. The study’s third hypothesis (H3) states that sports coaching impacts organizational climate. The result of this hypothesis shows that *t*-statistics = 11.15 with *p*-value = 0.000 (*p* < 0.05), thus this hypothesis has also been accepted. The study’s fourth hypothesis (H4) states that sports coaching impacts on career development. The result of this hypothesis shows that *t*-statistics = 1.61 with *p*-value = 0.108 (*p* < 0.05), thus this hypothesis has been rejected. The study’s fifth hypothesis (H5) states that organizational climate has an impact on academic development. The result of this hypothesis shows that *t*-statistics = 1.71 with *p*-value = 0.087 (*p* > 0.05), thus this hypothesis has also been rejected. The study’s sixth hypothesis (H6) states that organizational climate has an impact on career development. The result of this hypothesis shows that *t*-statistics = 16.32 with *p*-value = 0.000 (*p* < 0.05), thus this hypothesis has been accepted.

The study’s seventh hypothesis (H7) states that organizational climate mediates the relationship between social skill coaching and academic development. This hypothesis has been rejected as *t*-statistics = 1.57 with *p*-value = 0.116 (*p* > 0.05). The study’s last and eighth hypothesis (H8) states that organizational climate mediates the relationship between sports coaching and career development. This hypothesis has been accepted as *t*-statistics = 10.05 with *p*-value = 0.000 (*p* < 0.05).

## Discussion

This research focuses on the program curriculum for developing social skills and sports coaching in academic and career development under sports humanities and sociology. It yielded some interesting results. Similar investigations were carried out to evaluate the organizational methods in sports for obtaining the positive outcomes of the youth under YDPs. Moreover, the organizational climate was proved to be an essential component in directing the longitudinal relationships of organizational processes. The direct role of organizational climate was studied and some exciting results were reported by Lower-Hoppe et al. (2020) and indirect effects of it being a mediator were suggested by Ehrhart and Raver (2014). This study investigated direct impacts of coaching social skills on organizational climates, academic development, sports coaching effect on organizational climate, and career development. The mediating effects of organizational climate on academic and career development were also evaluated in this study.

This study was based on several hypotheses indicating organizational processes’ relationships, including coaching social skills and sport coaching on career and academic development. The first hypothesis evaluated the effect of coaching social skills on academic development. These kinds of relationship have been studied before and found significant impacts (Caprara et al., 2000). The first hypothesis was accepted and proved that coaching social skills had a significant impact on developing the student’s academic level. As discussed by earlier researchers, not only teaching academics help, but coaching for social skills also has positively impact on student’s cognition, ultimately improving the academic achievements. The second hypothesis was also accepted in this study as there was a direct relationship between coaching social skills and organizational climate. Coaching social skills is an organizational process that is a part of organizational climate improvement suggested by Newman et al. (2016) and Lower-Hoppe et al. (2020). Therefore, such results were obtained.

The third and fourth hypotheses were about the direct relationships of sports coaching with organizational climate and career development. The third hypothesis was accepted about the relation of coaching sports to students to improve organizational climate. Sports coaching is also an organizational process; therefore, it also leads to improving organizational climate. Similar results were obtained by Newman et al. (2016) and Lower-Hoppe et al. (2020). The fourth hypothesis was rejected as sports coaching that could not directly link with career development. The career development of athletes is directly related to sports coaching in different organizational setups as suggested by Shields and Bredemeier (2010) and Neely and Holt (2011). But, in the case of student’s coaching sports, there needs to be investigating more into career development.

The fifth and sixth hypotheses were about organizational climate and its direct relationships to career development and academic development. The relationship of organizational climate with academic development was rejected. In contrast, the relationship of organizational climate with career development was accepted as organizational development as mediator is a strong pillar and direct relations in case of students is a weak link; therefore, these results are obtained. It could lead to career development, but not to academic development. It also suggested that it needed a mediation for the relationship of organizational climate with the students’ academic development. These kind of relationships were suggested by Perry et al. (2005) and Lower-Hoppe et al. (2020).

The mediating role of organizational climate was also studied in this research, which suggested that direct relationship of coaching social skills for academic development did not need a mediation, as the direct hypothesis was also accepted and no need of such mediator was required between the relationship. While organizational climate proved to be a significant mediator between sports coaching and the career development. The direct connection was solely rejected as sports coaching that could not significantly impact the student’s career development. This result indicated a strong need between the two factors for mediation, as sports coaching is a part of organizational process and organizational climate significantly mediates the relationship. Such mediating role of organizational climate was studied before and yielded significant results (Mehralian et al., 2020).

## Conclusion

In the present era, the development of skills through coaching is considered influential among youth because skills are required to survive in this rapidly changing environment. Therefore, this study aimed to examine the role of program curriculum in building social skills and sports coaching in academic and career development under sports humanities and sociology. This study revealed that social skills coaching meaningfully impacted both the Chinese universities’ academic growth and organizational climate. This study also found a considerable influence of sports coaching on organizational climate, but highlighted no influence on the career development. Moreover, the organizational climate had a significant relationship with career development, but an insignificant association with academic development. Furthermore, organizational climate partially mediated the relationship between sports coaching and career development rather than between social skills coaching and academic development.

### Practical Implications

This study inculcates some practical implications for universities around the world. First, the universities must hire a highly competent coach to provide training and coaching to the students to develop their social skills and sports orientation. The coach’s competency must be measured using effective methods such as physical tests, preassessments, and psychological tests. Second, the universities in China have to develop a culture of social skills and sports to improve academic and career development. Third, sports festivals must be made common in Chinese universities, so that the organizational climate can be improved and the students can have better career development. However, this study includes some limitations that can be considered for future studies. This study took data from only members of the sports federation enrolled in different programs in Chinese universities; therefore, future studies can examine other populations such as European countries. Moreover, the future studies can use other mediating variables such as personal growth or psychological well-being to examine its effect on the existing model.

### Limitations and Recommendations

By measuring the role of social skills coaching on career and academic development, this study has certain limitations. First, it has used the purposive sampling technique; however, stratified random sampling could better present the results according to each department. Furthermore, this study has been confined to the variables of interest; nonetheless, more objective variables can be included in the framework for future studies. For example, future studies can collect data from the staff or coaches to understand the study’s long-term results. Third, this study has used the adapted scales. However, future studies can develop their scales following the coaches or staff level sample accordingly. This study has focused on the sports-oriented students’ goals. Future studies may focus on the consequences of social skills and sports coaching to further understand these variables under sports leadership.

## Data Availability Statement

The original contributions presented in the study are included in the article/supplementary material, further inquiries can be directed to the corresponding author.

## Author Contributions

JY conceived and designed the concept. ZJ collected the data and wrote the manuscript. Both authors read and agreed to the published version of the manuscript.

## Conflict of Interest

The authors declare that the research was conducted in the absence of any commercial or financial relationships that could be construed as a potential conflict of interest.

## Publisher’s Note

All claims expressed in this article are solely those of the authors and do not necessarily represent those of their affiliated organizations, or those of the publisher, the editors and the reviewers. Any product that may be evaluated in this article, or claim that may be made by its manufacturer, is not guaranteed or endorsed by the publisher.
